# Human Ovarian Cortex biobanking: A Fascinating Resource for Fertility Preservation in Cancer

**DOI:** 10.3390/ijms21093245

**Published:** 2020-05-04

**Authors:** Erica Silvestris, Giuseppe De Palma, Stefano Canosa, Simone Palini, Miriam Dellino, Alberto Revelli, Angelo Virgilio Paradiso

**Affiliations:** 1 Gynecologic Oncology Unit, IRCCS Istituto Tumori “Giovanni Paolo II”, 70124 Bari, Italy; ericasilvestris85@gmail.com (E.S.); miriamdellino@hotmail.it (M.D.); 2 Institutional BioBank, Experimental Oncology and Biobank Management Unit, IRCCS Istituto Tumori “Giovanni Paolo II”, 70124 Bari, Italy; g.depalma@oncologico.bari.it; 3 Gynecology and Obstetrics 1, Physiopathology of Reproduction and IVF Unit, Department of Surgical Sciences, S. Anna Hospital, University of Torino, 10042 Torino, Italy; s.canosa88@gmail.com (S.C.); alberto.revelli@unito.it (A.R.); 4 IVF Unit, Cervesi Hospital Cattolica, Cattolica, AUSL Romagna, 47841 Cattolica, Italy; simonepalini@yahoo.it

**Keywords:** cryopreservation, gonadotoxicity, oncofertility, ovarian cortex, ovarian stem cells, tissue biobanking

## Abstract

Novel anti-cancer treatments have improved the survival rates of female young patients, reopening pregnancy issues for female cancer survivors affected by the tumor treatment-related infertility. This condition occurs in approximately one third of women of fertile age and is mainly dependent on gonadotoxic protocols, including radiation treatments. Besides routine procedures such as the hormonal induction of follicular growth and subsequent cryopreservation of oocytes or embryos, the ovarian protection by gonadotropin-releasing hormone (GnRH) agonists during chemotherapy as well as even gonadal shielding during radiotherapy, other innovative techniques are available today and need to be optimized to support their introduction into the clinical practice. These novel methods are hormone stimulation-free and include the ovarian cortex cryopreservation before anti-cancer treatments and its subsequent autologous reimplantation and a regenerative medicine approach using oocytes derived in vitro from ovarian stem cells (OSCs). For both procedures, the major benefit is related to the prompt recruitment and processing of the ovarian cortex fragments before gonadotoxic treatments. However, while the functional competence of oocytes within the cryopreserved cortex is not assessable, the in vitro maturation of OSCs to oocytes, allows to select the most competent eggs to be cryopreserved for fertility restoration.

## 1. Introduction

Infertility has been recently assumed not only as a medical condition but also as a social disease, which appears dependent on a series of pathogenic events sometimes related to the modern lifestyle, since it is estimated to affect around 186 million people worldwide [1]. Besides the healthy couples who experience chronic failure to conceive, young oncologic patients are also at high risk for infertility which is primarily dependent upon adverse effects of anti-cancer treatments and is also frequently permanent after the malignancy healing. In fact, since modern cancer treatments have undoubtedly improved the therapeutic results and may lead to complete remission of the disease in a large number of cases, in female cancer survivors under 45 years the quality of life may be fully restored only when pregnancies are possible [2]. Fertility preservation (FP) in oncologic patients is thus to be considered today as a topic of primary importance.

Indeed, as a result of chemotherapies, radiotherapy, innovative treatments, or their combination, a premature, significant depletion of the ovarian reserve (OR) may occur in at least one third of the female cancer population, leading to a condition of permanent infertility [3,4,5]. To this, the international guidelines for FP in oncology recommend an early personalized counseling for all patients in reproductive age to screen if they are suitable candidates for FP programs in relation to the evaluation of their OR. Therefore, multidisciplinary counseling among different specialists may thus be functional in the management of young oncological female patients in order to preserve and restore their fertility before gonadotoxic treatments and after their conclusion, respectively.

In this regard, several fertility preservation options ranging from routine to experimental strategies are now available to counteract the treatment-related infertility risk. Besides the gonad shielding during radiotherapy and the ovarian suppression by gonadotropin releasing hormone agonist (GnRHa) [6], both the American Society of Clinical Oncology (ASCO) and the European Society for Medical Oncology (ESMO) recommend the oocyte and embryo cryopreservation before anti-cancer treatment as the mostly employed procedures able to guarantee motherhood in post-puberal female cancer survivors [7,8]. These techniques require a controlled ovarian stimulation (COS) by gonadotropin injections for at least 12 days to support the multiple follicular growth (MFG), in association with repeated ultrasound examination and the evaluation of hormonal biomarkers such as estradiol. After the ovulation induction, the retrieved oocytes are morphologically evaluated and cryopreserved as mature eggs by slow-freezing or vitrification procedures [9], or can be fertilized in vitro and then stored as embryos [8]. Although these cryopreservation procedures appear effective in terms of successful pregnancies in healthy women [10], the preparative COS is not suitable in cases of pre-puberal girls as well as in circumstances requiring urgent anti-cancer treatments as for hematologic malignancies which cannot be retarded in relation to the time necessary to induce MFG. Furthermore, the additional estrogen-related oncogenic risk in cases of estrogen-sensitive cancers such as breast or endometrial tumors [11] renders COS not completely safe. To overcome these limitations, however, oogonial cells may be retrieved as immature oocytes and, after their in vitro differentiation to mature eggs, cryopreserved with the final purpose of avoiding the delay of urgent anti-cancer treatments [12].

An experimental ‘safe’ procedure independent from COS and menstrual cycle phases is the cryopreservation of ovarian cortex fragments and their subsequent reimplantation after cancer healing. This method appears suitable for both prepuberal and adult patients with functional OR, whose ovarian biopsies are easily obtainable by laparoscopic or laparotomic surgery without COS and even in conditions of anti-cancer treatment urgency. Within 24 hours, ovarian fragments are surgically retrieved and cryopreserved using either slow freezing or vitrification [13]. Despite the advantages of this technique and its suitability if compared to other FP methods, there are a few underestimated aspects concerning the frequently inadequate pool of mature oocytes, the viability of eggs after thawing, as well as the risk of implanting residual malignant cells originally resident in the frozen ovarian cortex, such as leukemia cells [14].

In this context, the recent discovery of the ovarian stem cells (OSCs), first in animal models and then in the human ovarian cortex, provided a novel approach potentially suitable to achieve FP in oncological patients without the previously described concerns [15]. Once isolated from the ovarian cortex and cultured in vitro, OSCs manifest an intrinsic potential to differentiate into mature oocyte-like cells (OLCs) [16], thus providing new possibilities to replace hormone balancing in menopause as well as to treat premature ovarian insufficiency (POI) induced by anti-cancer treatments and, eventually, for further conditions causing female infertility.

Here, we revise the FP options for female cancer survivors focusing on the currently adopted procedures as well as on innovative methods that could be prospectively proposed.

## 2. Ovarian Structure and Components

Human ovaries originate from the intermediate mesoderm of the developing embryo. By week four of gestation, the endodermal pluripotent cells generate the primordial germ cells (PGCs), that will become the female germ cells [17]. The mechanisms underlying PGCs differentiation are not completely elucidated and several bone morphogenetic proteins (Bmp) such as Bmp2 [18], Bmp4 [19,20,21] and Bmp8b [20] are functionally involved in mammals. At the fifth week, PGCs proliferate by mitosis and migrate to the gonadal ridge where they become oogonia [22,23], which actively replicate reaching a peak of six to seven million cells at the 20th week [23] when they are surrounded by a layer of follicular cells, namely the granulosa, thus forming the primordial follicle [24]. 

Germ cells gradually undergo a numeric decrease due to both apoptosis and follicle atresia which provide the final oocyte pool resulting of approximately one to two million cells at birth, 300,000 cells at puberty [25,26] and approximately 400 mature oocytes during the woman’s reproductive lifespan [27] until the menopause [28,29].

**Figure 1:**
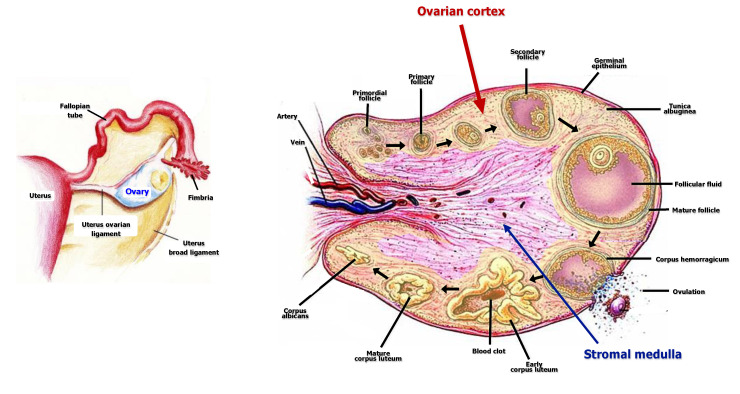
Anatomic location and structure of the ovary including cortical components.

A critical aspect of the age-related gradual decrease of the OR is the widespread opinion that primordial follicles cannot be physiologically replaced during the woman’s reproductive life. This dogma has been recently revisited in relation to the discovery of a population of OSCs capable of generating new oocytes both in mice and in in vitro human models, although the physiological role of these cells has still to be elucidated [30]. OSCs are located within the ovarian cortex, which constitutes the anatomic site containing the major source of cells as hormones, soluble factors and structural components required for mammalian oogenesis. In a recent study by Woods et al., indeed, it has been hypothesized that the differentiation of OSCs to competent oocytes is directly supported by somatic cells surrounding them in cortical niches [31] (Figure 1).

The cortex is the most organized tissue in the ovary and includes many follicles defined in their developmental as primordial, primary, secondary and tertiary. The follicular cell layers, separated from the oocyte by the zona pellucida, differentiate into granulosa cells (GCs) at the first stage, namely the primary follicle, while stromal components of the ovary organize themselves into concentric layers around the granulosa cells, forming the theca [32]. The granulosa in the secondary follicle becomes multilayered and surrounds the oocyte while producing the follicular fluid within the antrum, thus originating the cumulus oophorus inside antral follicles. Then, theca cells activate their steroidogenesis and their products are converted to estrogens by granulosa cells.

Cha et al., reported that a subset of immature cells which proliferate together with oocytes within the developing follicles, namely undifferentiated granulosa cells (UGCs), also concur to regulate the follicular final maturation resulting from their expression of stemness genes and related markers [33]. To support this, other investigators have expanded a somatic cell population showing stemness genes as POU domain class-5 transcription factor-1 (Pou5f1) and Nanog from the ovarian cortex. These cells exhibit a typical UGC molecular pattern as both Wingless-type MMTV integration site family member 4 (Wnt4) genes, Forkhead box L2 (Foxl2) and Follicle stimulating hormone receptor (FSH-R). These data suggested that granulosa stem cells as UGCs are functional during the ovarian life and are perhaps implicated in supporting oogenesis and probably the OSC differentiation [4].

## 3. Current Techniques Using Cryopreserved Ovarian Cortex

Ovarian tissue cryopreservation (OTC) is a novel and alternative option for FP in female cancer patients who urgently need chemotherapy, in particular with neoadjuvant protocols [34,35]. The retrieval of ovarian cortex fragments is slightly invasive and may also be applied to patients with endometriosis [36], Turner’s syndrome [37], or to pre-pubertal girls with cancer [38].

Besides avoiding the COS by gonadotropin injections to support the MFG usually adopted for the oocyte/embryo cryopreservation, the major advantage of using cryopreserved cortex includes its feasibility independent from the menstrual cycle, both in pre-pubertal and adult cancer patients with adequate OR previously evaluated, to acquire sufficient viable follicles from each cortical fragment.

The first case of successful human ovarian tissue transplantation was reported by Oktay et al., [39] nearly 20 years ago. Subsequently, Donnez et al., [40] described the first live birth after auto-transplantation of cryopreserved ovarian tissue in humans, and since then it is estimated that more than 10,000 girls and women worldwide have undergone OTC resulting in more than 130 healthy newborns [41,42,43]. Published data are reported in Table 1 which summarizes both the pros and cons of major FP procedures, including OTC that, however, is considered still experimental [44].

**Figure 2:**
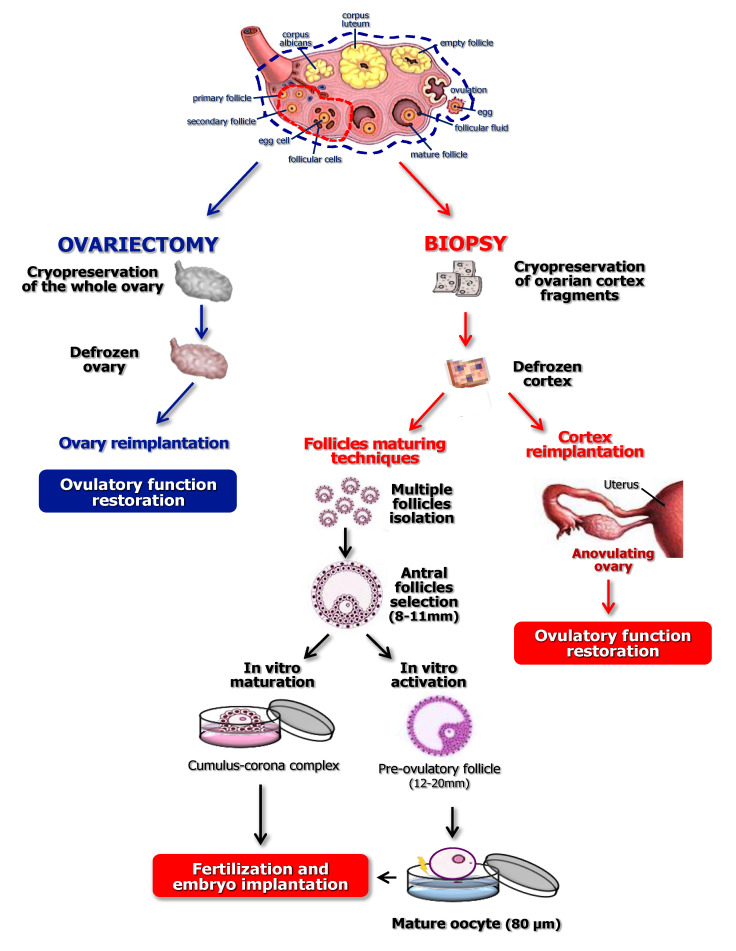
Potential applications of whole ovary and cortex fragments cryopreservation and transplantation for fertility preservation.

As depicted in Figure 2, at present there are three main options adopting the reimplantation of cryopreserved ovarian tissue [45]: i) cortical pieces obtained from one or both ovaries replanted either orthotopically or heterotopically [46]; ii) isolation and in vitro maturation (IVM) or activation (IVA) of the recruited follicles using scaffolds [47,48]; and iii) whole ovary reimplantation with vascular anastomosis [49,50]. Ovarian biopsy is usually performed by laparoscopy and cortical fragments of approximately 5 mm are exposed to freezing solutions and stored in liquid nitrogen. Permeability to cryoprotectants is essential to obtain viable oocytes after thawing and thin fragments are probably better perfused. The oocyte content is related to the patient’s age and is expectedly consistent in girls.

Besides the simple OTC procedure, however, several drawbacks need to be addressed. The first is related to the low number of viable oocytes after thawing the cortical fragments. In fact, removal of the ovary from blood and oxygen supply, processing of the cortical tissue, freezing–thawing protocols and transplantation may affect the survival of the follicle pool [51,52]. Freezing–thawing procedures, indeed, lead to follicular pool decrease due to ice crystal formation which can induce cell injury in both cytoplasmic organelles and cell membrane [53]. Thus, it is essential that OTC is primarily performed by very slow freezing and quick thawing procedures to preserve the oocytes from the formation of intra-cytoplasmic ice crystals [54]. On the other hand, unsuccessful OTC grafts are also dependent on the follicular loss induced by early ischemia after transplantation, which is strongly related to the oocyte cumulative injury promoted by biopsy, cryopreservation, defrosting of cortical pieces and surgical reimplantation [55].

Cryopreservation of a whole ovary followed by vascular anastomosis of the ovarian pedicle has been suggested to decrease ischemic injury and provide a suitable follicular reserve as well as longer lifespan of the transplant [56,57,58]. This procedural variant of OTC has been performed in a small cohort of patients and, although providing encouraging results in humans [49,50], definitely requires further studies, particularly in optimizing the freezing protocols, before its introduction into the clinical practice.

Another variant is the implantation and grafting of isolated ovarian follicles. Dolmans et al., showed that isolated human follicles survive and grow for one week after xenograft using plasma clots in mice [47]. Further, Amorim et al., observed that small pre-antral follicles from frozen–thawed tissue survive in vitro and increase their size after seven days of incubation in a three-dimensional matrix of alginate hydrogel [48]. Although preliminary, these results support the in vitro culture of isolated follicles from defrosted ovarian cortex as a suitable alternative to use of cryopreserved ovarian tissue since it is also possible to select the follicles to be isolated and cultured in vitro. However, this procedure is not free from potential damages deriving from both cryopreservation and thawing of the ovarian biopsy fragments.

To optimize the freezing strategies, vitrification has been proposed as an alternative procedure. This method is based on the induction of an ultra-rapid cooling process using high concentrations of cryoprotectants, and is apparently functional in preventing cell injuries by producing a glass-like amorphic state of cells, as well as in maintaining also stromal morphological and structural integrity similar to fresh tissue [59,60,61]. Furthermore, Diaz-Garcia et al., evaluated the live birth rate following oocyte vitrification with respect to OTC and transplantation in oncological patients undergoing gonadotoxic treatments, and reported that that despite slightly higher results were achieved after oocyte freezing, the vitrification would represent an alternative option for natural pregnancies when oocyte cryostorage is not feasible [62].

More recently, utilization of slush nitrogen has been proposed to improve vitrification efficacy since morphology, ultrastructure and viability of both follicles and stromal cells are apparently better preserved in defrosted components, as compared with the same procedure using liquid nitrogen [63,64].

A further variant includes the IVM of immature oocytes aspirated just before the ovary specimen freezing and cryostorage [65], that would be suitable for next use once maturated in vitro. Although preliminary studies encourage this approach in fertility restoration programs, it appears inadequate in cancer patients also for the risk of cancer relapse [66,67] induced by the hormonal stimulation in patients with hormone sensitive cancers. In fact, the reintroduction of cancer cells with ovarian tissue transplantation appears as a major drawback in patients with hematologic malignancies such as acute leukemias, in which circulating leukemic cells are present in cryopreserved ovarian biopsies and can cause reappearance of the disease after the procedure. Several reports definitely describe this risk in patients with leukemia and lymphomas [68], for which autologous OTC and reimplantation is not recommended in their FP program.

Finally, an ultimate addition to OTC has been recently proposed, namely the in vitro activation of dormant follicles (IVA), to increase the number of mature oocytes promptly originated after transplantation [69]. The procedure includes two steps after tissue thawing: the first is based on the complete fragmentation of cortex biopsies in order to promote follicle growth and, at the same time, the progression from secondary to early antral stage by disrupting the Hippo signaling pathway [70]. The second step involves the in vitro culture of cortical pieces with a mix of both PTEN inhibitor and PI3K activator for 48 hours, in order to stimulate the activation of dormant primordial follicles [71]. This procedure has been reported by Suzuki et al., who successfully restored fertility in patients diagnosed with POI after auto-grafting of vitrified human ovarian tissue coupled with follicle obtained by the IVA method [72]. Considering the significant reduction of the ovarian lifespan and reproductive potential of this approach, this fascinating strategy should be proposed mainly to women undergoing OTC and reimplantation in more advanced fertile age [69].

Although these PF practices allow cancer patients to restore their fertility after the cancer healing, there are still some limitations regarding their applicability. At present, in fact, it is not yet known whether or not the efficacy of these procedures in cancer patients is comparable with the results obtained in the healthy infertile population that have undergone the same treatments. On the other hand, based on the poor literature in this regard it cannot be assumed that specific FP methods should be separately suggested to defined histotypes of cancers. However, despite these unsolved questions it appears preferable to avoid COS in hormone-dependent tumors as well as OTC in hematological malignancies to prevent additional oncogenic risks of these procedures. 

**Figure 3:**
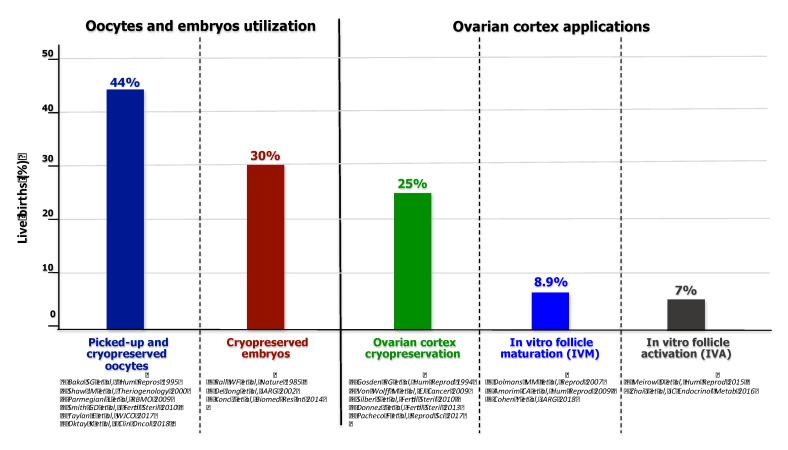
Live birth rates obtained by traditional oocyte pick-up and cryopreservation (left) and novel techniques using ovarian cortex biopsies (right), as reported in literature.

In conclusion, the OTC and reimplantation technique appears as a suitable method for FP in cancer patients, obtaining a variable percentage of outcomes which reach approximately 25% of live births [73]. Figure 3 depicts the rate of live births by conventional procedures and utilization of cryopreserved ovarian cortex.

However, although this FP method is independent from hormonal COS, menstrual timing and is suitable for both pre-pubertal and adult patients with adequate OR, it is not free of applicative restrictions, which prevalently include the impossibility to select the most competent eggs to be fertilized, as currently accomplished by oocyte retrieval in infertility centres, and the risk of malignant cells reimplantation, as occasionally experienced in hematologic malignancies [68].

## 4. The Ovarian Cortex Transplantation to Prevent the Cancer Treatment Induced Infertility

Ovarian cortex cryopreservation is a suitable technique in pre-pubertal girls with cancer since ovarian stimulation and in vitro fertilization are not feasible in these patients [74], implantation of autologous cryopreserved cortex may functionally restore oogenesis and reproductive function [75]. The original application included the implantation of a fresh ovary into the forearm with vascular anastomosis in a lymphoma patient undergoing pelvic irradiation [76], whereas in subsequent clinical studies the follicular growth was also observed after implanting ovarian fragments without vascular anastomosis [77]. The earliest case of ovarian restoration using cryopreserved human ovarian fragments was reported in 2000 [39], although the first live births were obtained a few years later and supported the efficacy of this procedure in fertility recovering [40,46,78].

In general, after thawing the fragments are implanted into residual ovaries or into a pelvic peritoneum pocket within the ovarian fossa (orthotopic sites), or at distance from the ovary, at subcutaneous sites (heterotopic sites). However, while follicular growth has been observed at both orthotopic and heterotopic sites, almost all pregnancies and live births have been reported after transplantation at an orthotopic site, which continues to be the first choice when applicable [43,79]. Indeed, there are only a few reports describing successful pregnancy and live births also in replanting the cryopreserved ovarian tissue in heterotopic sites [80,81,82]. At present, however, more than 130 babies were born worldwide by using cryopreserved tissue and among them only very few resulted from vitrified tissue [72], leading to intensive debate on the most fruitful method to be applied [83].

It is estimated that following the procedure of cryopreserved ovary transplant, the functional recovery rate exceeds 90%, with a live birth rate per patient ranging from 18.2% to 40% in the literature [62,84,85,86]. However, differences detected between centers are likely due to the small cohorts of patients enrolled in single studies, which also included differently aged female subjects since it has been observed that the ovarian tissue cryopreserved before puberty or menarche would lead to better results even when transplanted in advanced age [87,88].

Despite the evidence of this favorable outcome, the debate is today open whether or not to define the procedure of ovary cryopreservation and transplantation as experimental. Ovarian tissue cryopreservation is no longer considered experimental in Israel, while in Europe and USA its utilization still needs to be regulated; almost all young cancer patients have been treated following dedicated research protocols selecting those at high risk of gonadotoxicity related to radiation treatments, chemotherapy and stem cell transplantation conditioning [89,90].

As mentioned, this procedure is at risk of re-introducing cancer cells. However, this risk is variable in relation to the malignancy and its location, resulting higher risks in leukemia, Burkitt lymphoma, neuroblastoma and ovarian cancer [91,92]. Also, it has been demonstrated that human ovarian tissue containing cancer cells may transfer the disease into mice after xenograft [68]. By using molecular approaches, several investigators demonstrated that more than 30% of the ovarian specimens from patients with chronic myeloid leukemia, as well as 70% from acute lymphoblastic leukemia patients, included variable amounts of leukemic cells. To prevent this risk, the ovarian tissue harvested during the complete remission following first-line chemotherapy should be free of cancer cells and thus suitable to be cryopreserved for future transplantation as shown in experimental xenograft models [93,94].

To reduce the cancer reimplantation risk, purging of tumor cells [95], isolation of follicles for preparing disease-free follicle suspensions to be grafted as artificial ovary [96,97,98] and in vitro follicular culture systems have been proposed [99]. Theoretically, the artificial ovary is a short-term substitute of the natural ovary in which isolated follicles, ovarian stromal and endothelial cells in combination with growth factors can be encapsulated within a biomaterial replacing the natural extra-cellular matrix architecture before its surrounding cells undergo settlement within a new functional organ. This novel application, however, needs to be fully investigated before its translation to the use in FP programs.

In contrast with the full ovary transplantation, the ovarian fragments implantation is performed without vascular anastomosis and usually the number of primordial follicles decreases over 50% for the concurrent tissue ischemia. The follicular depletion may thus significantly decrease the graft lifespan. Once the neoangiogenesis is re-established, the lifespan of the tissue graft is variable, ranging from less than 1 year to more than 10 years, and prompt neovascularization is apparently accelerated by intense granulation within the transplantation site [79,100]. Therefore, it has been proposed that a preliminary preparation of a peritoneal compartment one-week before the ovarian cortex reimplantation may better support the neovascularization of fragments [40], whereas utilization of extracellular tissue matrix would also produce a similar effect [101]. However, the benefit of these procedures has not been established.

In more than 70% of women aged below 40 years with POI, residual ovarian follicles are detectable, although in a dormant state. Thus, utilization of this resource has also been investigated and the isolation of dormant follicles followed by their IVA has recently resulted in efficient follicle growth detected in recipient ovaries as early as 20 days after the fragments’ implantation within the cortical ovarian tissue [102,103].

Based on these considerations, the OTC and reimplantation procedure for FP includes both advantages and drawbacks, particularly in oncology. Whilst it has been improved in technical methodology for acquiring viable follicles as well for improving the grafting of implanted ovarian biopsies, on the other hand the selection of follicles is based only on their ultrasound detection and the quality of oocytes to be replanted is usually not investigated. Although the risk to regenerate certain tumors in relation to the presence of cancer cells in the cryopreserved ovarian cortex can be limited by selecting the best time for the biopsies, in which the tumor remission is evident, the procedure cannot be considered completely safe at least in patients with hematologic neoplasias and some solid cancers.

## 5. Ovarian Stem Cells (OSCs): A Novel Resource of the Ovarian Cortex

The discovery of functional adult stem cells named OSCs in ovarian cortex mammals has recently revisited the paradigm of a fixed pool of oocytes, offering new therapeutic options in female infertility also related to cancer treatment, in order to reduce risk of POF by the stemness technology. Their identification, indeed, is in contrast with the central dogma in reproductive sciences that inherited numbers of oocytes are not subjected to renewal after birth.

Previous studies proved the existence of OSCs in rats [16], pigs [104], non-human primates [105] and in mice [106] by the expression of DEAD box polypeptide 4 (Ddx4) [107], a transmembrane germline marker of oogonial differentiation which internalizes its COOH tale during final oocyte maturation, in combination with other stemness markers (Fragilis, Stella, OCT4, SSEA4) [108].

**Figure 4:**
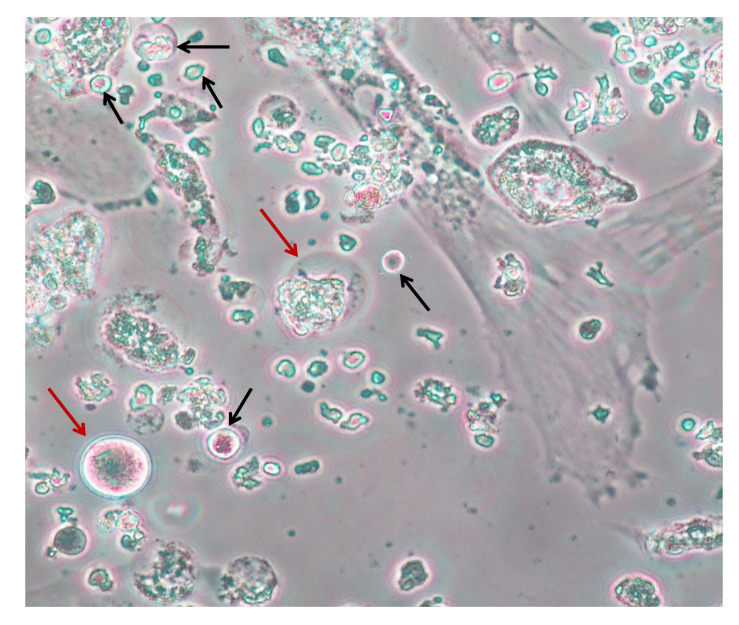
In vitro maturation of ovarian stem cells (OSC)s to oocyte-like cells (OLCs) of large (red arrows) and small (black) size in three weeks cultures.

Similar studies in women showed that OSCs were detectable also in ovarian cortex fragments from healthy women both in pre and post-menopausal age [109]. The authors isolated the OSCs by immunomagnetic separation by anti-human Ddx4 reagents, and generated cultures of the Ddx4^+^ cell population also assessed for both FRAGILIS and SSEA4 markers. Their expansion in culture resulted in large oocyte like cells (OLCs) sized up to 80 μm of diameter, whose intra-culture percent values were almost comparable in cultures from menopausal and non-menopausal women. The OLCs subsequently sorted by DEPArray methodology and investigated in their oocyte differentiation by droplet digital PCR (ddPCR) revealed equivalent levels of *GDF9* and *SYCP3* mRNA as finally expressed genes in both pre- and post-menopausal patients, thus supporting their final differentiation state of mature oocytes [109]. By contrast, a small Ddx4^+^ cell population expressing *DPPA3* mRNA, a marker of immature oogonial cells undetected on OLCs, were also detected in the majority of cultures. Finally, the progression of meiosis in haploid cells was also demonstrated by both DNA content [110] and FISH analysis, revealing single signals on chromosome X and 5 [109] in terminally differentiated large OLCs (Figure 4).

Recently, Telfer and colleagues, showed that the reimplantation of OLCs in mice sterilized with busulan resulted in oocyte repopulation of ovaries leading to fertilization and pregnancies [111], thus restoring the fertility by oocytes derived from OLCs. Thus, based on these data, there is enough evidence that oocytes differentiated by human OSCs appear competent in vitro and these properties need to be better ascertained for their future in vivo use also in humans. However, to obtain expanded populations of oocytes it is necessary to pursue the investigative aspects as the identification of specific oogenic factors acting during the final differentiation stage to mature eggs.

In this contest, UGCs capable to interact with newly generated oocytes and form primordial follicles are suspected by Akahori et al., to prime the final oocyte maturation for their expression of stemness markers and genes [4]. However, this appears controversial since UGCs probably derive from pluripotent stem cells (PSC) which are resident in the ovaries and are inducible in vitro [112]. Thus, in search of a model improving OSC differentiation to mature oocytes particularly in cancer-associated infertility, several investigators as Anchan et al., suggest the utilization of patient-derived induced-PSCs to generate autologous granulosa cells to induce aggregation of OSCs and OLCs with the purpose of expanding the folliculogenesis in vitro and selecting the best mature oocytes [113].

Despite that the preliminary results on OSCs are promising, the stemness technology needs to be further investigated and additional studies are needed in order to demonstrate the suitability of these cells in humans and clarify the scientific scepticism on their potential to restore the oogenesis with the purpose of guarantee its standardization and clinical application as a safe technique to restore fertility also in oncological patients. In this specific field, the OSCs would become of primary interest since they will be probably able to restore fertility in young patients without the hormone conditioning with the additional advantage of exploring the quality of the eggs usable for FP thus acquiring the characteristics of safe and selective procedure.

Another type of SCs is represented by the inducible pluripotent stem cells (iPSCs), which are generated by the genic reprogramming of somatic cells into a pluripotent state. Like embryonic stem cells, they are able to self-renew and differentiate. Scientists have engineered these iPSCs by transfection in human somatic cells of four transcription factors, namely Nanog, Sox2, c-Myc and Klf4, capable to revert the differentiated cells to embryonic-like state. Since these newly generated cells show the morphology, pluripotency and capacity to generate teratomas like ESCs, the authors defined these cells as “induced pluripotent stem cells”. However, while the ability to develop iPSCs from differentiated somatic cells is quite affordable and exciting, the system has two major drawbacks. Firstly, the reprogramming efficiency is low, suggesting that inside the cell there may be mechanisms that prevent the reprogramming process; secondly, there is the oncogenic potential of iPSCs, as reflected in their ability to form teratomas in mice. Thus, this revolutionary topic in the field has provided the clinical and scientific communities a second tool for cell-based therapy [114].

## 6. Potential Utilization of OSCs for Female Fertility

Results from the basic research on OSCs open a direct issue on the safest FP procedure to be suggested to young oncological female patients before any gonadotoxic treatment, since utilization of OSCs in oncologic female patients may provide more advantages with respect to other procedures currently applied to restore the ovarian reserve. Oocyte and embryo cryopreservation is largely proposed to patients as the most successful procedure to obtain pregnancies after healing the cancer, but the potential risk to stimulate the tumor by the hormonal burst with estrogens is commonly under-evaluated and not well assessed in clinical studies. There are only few reports claiming this risk [115], which is frequently ignored in planning FP programs in women with gynecologic cancers for whom the ovarian suppression by GnRH agonists is apparently risk-free as compared to the estrogen stimuli [4]. On the other hand, the few week-time period needed to induce MFG makes oocyte cryopreservation sometimes inappropriate for patients immediately requiring neoadjuvant treatments.

Ovarian cortex cryopreservation and re-implantation should be undoubtedly considered a procedure free of oncogenic hormone-induced risks, but the related drawbacks have been already commented on in this article, and primarily concern the suitability of eggs located within the cryopreserved ovarian cortex. The lack of a direct control on oocyte population before implanting the cryopreserved cortex, the unpredictable results in relation to the high variability of successful outcomes, as well as the possibility that in selected tumors the grafted biopsy specimen may include malignant cells capable to regenerate cancer, are topics of interest to be solved in the near future [92].

By contrast, the OSC technology would overcome these risks and restrictions. Furthermore, in patients requiring urgent anti-cancer treatments, preventive laparoscopic biopsy of the ovarian cortex would allow the isolation of OSCs with subsequent differentiation and expansion in vitro of a large population of OLCs from which the highest quality and suitable cells could be selected for freezing and cryostorage. The in vitro expanded OLCs are obtainable independently from the hormone stimuli as well as from the menstrual phases, and their cryopreservation is based on the same protocols adopted for the hormone-stimulated MFG and eggs recruitment. Overall, in contrast with the ovarian cortex cryostorage and reimplantation, this practice allows to select the most viable oocytes to be cryopreserved as well as to store high numbers of oocytes easily available for each patient.

In the regenerative medicine era, investigation of the OSC properties applied in the FP in oncology is a very interesting field of clinical research and in our research institution we have in progress a similar project in young oncologic patients undergoing abdominal surgery before gonadotoxic treatments, with the purpose to identify the oogenic factor(s) involved in the final differentiation of OLCs to mature eggs and optimize this technology for future translation to clinical use.

In conclusion, the OSC technology needs to be intensively investigated with the aim to offer to all oncological patients undergoing anti-cancer treatments not only the safest method to avoid hormonal stimuli capable of influencing the tumor progression or the replant of malignant cells, but also the possibility to obtain in vitro and select the best oocytes for well-controlled fertility programs.

## 7. Conclusions

At present, the standard of care for young female cancer patient FP is the cryopreservation of mature eggs and preimplantation embryos, which sometimes are not feasible procedures, due to the need for urgent anti-cancer therapies or the personal history of a hormone-sensitive malignancy. Similarly, other alternative and safer FP strategies include the support of the biobank for the cryopreservation of ovarian cortex fragments for autologous transplantation, as well as for the storage of immature oocytes for in-vitro maturation and fertilization, once the anti-cancer treatments have been completed, with the aim to improve these methods in order to achieve a live birth rate comparable to that obtained by traditional oocyte and embryo cryopreservation. 

In this context, the ovarian cortex sampling and processing for Ddx4^+^ OSC isolation might also enable their subsequent in-vitro maturation to OLCs for in-vitro fertilization procedures. This technology, similarly to other FP strategies, also requires the biobank for the cryostorage of the most viable oocytes derived from Ddx4^+^ OSCs to be fertilized and implanted in uterus. Based on these innovative applications in terms of stemness in FP, it is desirable that this technique may undergo rapid optimization and, hopefully, approval by competent authorities to consent expected maternity projects in female patients who survived cancer but maintain the cancer treatment-induced infertility.

## Figures and Tables

**Table 1:** Schematic description of advantages and disadvantages of the major procedures adopted for fertility preservation (COS: Controlled ovarian hyperstimulation, OR: ovarian reserve).

FP Procedures	Age	Pros	Cons	References
Oocyte cryopreservation	-Postpuberal women	-Well established technique -High percentage of success	-Need for COS and cycle dependence -Need to delay oncological treatment -Oncogenic risk for hormonal-cancers -Not applicable in females with poor OR	[7]. Loren AW et al, 2013. [9] Parmegiani L et al, 2009. [10]. Annan JJ et al, 2013
Embryo cryopreservation	-Postpuberal women	-Well established technique -Good percentage of success	-Need for COS and cycle dependence -Need to delay oncological treatment -Oncogenic risk for hormonal-cancers -Not applicable in females with poor OR -Limited to few countries	[8]. Peccatori FA et al, 2013. [10]. Annan JJ et al, 2013. [11]. Bianchii V et al, 2012
Ovarian cortex cryopreservation	-Prepuberal women -Postpuberal women	-Immediate application -No need for COS or cycle dependence -No oncogenic risk for hormone sensitive cancers	-Experimental technique-Pelvic surgery -Oncogenic risk after replantation -Variable risk of unsuccess due to the oocyte depletion in implanted fragments -No possibility to select most viable oocytes -Limited to expert infertility centers	[34]. Jeruss J et al, 2009. [35]. Andersen C et al, 2019. [44]. Martinez F et al, 2015

## Abbreviations

FP	fertility preservation
OR	ovarian reserve
GnRHa	gonadotropin releasing hormone agonist
COS	controlled ovarian stimulation
MFG	multiple follicular growth
OSCs	ovarian stem cells
OLCs	oocyte-like cells
POI	premature ovarian failure
PGCs	primordial germ cells
Bmp2	bone morphogenetic protein 2
GCs	granulosa cells
UGCs	undifferentiated granulosa cells
Wnt4	Wingless-type MMTV integration site family member 4
Foxl2	Forkhead box L2
FSH-R	Follicle stimulating hormone receptor
OTC	Ovarian tissue cryopreservation
IVM	in vitro maturation
IVA	in vitro activation
Ddx4	DEAD box polypeptide 4
ddPCR	droplet digital PCR
PSC	pluripotent stem cells
